# The knowledge of Polish medical students about surgical treatment of obesity

**DOI:** 10.1007/s10353-015-0331-y

**Published:** 2015-07-09

**Authors:** M. Matłok, M. Pędziwiatr, P. Major, M. Nowakowski, M. Rubinkiewicz, M. Wyleżoł, P. Budzyński, A. Budzyński

**Affiliations:** 1 2nd Department of General Surgery, Jagiellonian University Collegium Medicum, Kopernika 21 Street, Cracow, Poland; 2 Department of Medical Education, Jagiellonian University Medical Collegium Medicum, Sw. Łazarza 16 Street, Cracow, Poland; 3 Department of Surgery, Military Institute of Aviation Medicine, Krasińskiego 54/56 Street, Warsaw, Poland

**Keywords:** Bariatric surgery, Metabolic surgery

## Abstract

**Background:**

Surgical treatment of morbid obesity is becoming an increasingly important approach for the treatment of this condition. However, knowledge about the possibility of surgical procedures among general practitioners is far from satisfactory. The source of the problem might be due to a lack of information about bariatric surgery in university curriculum.

**Methods:**

We assessed the knowledge of students from four Polish medical universities. The survey was conducted among 468 students, in their sixth (final) year of study. The survey included two parts—the first nine questions assessed of the level of the students’ knowledge about the methods of surgical treatment of obesity, and the following three questions allowed for an evaluation of the amount of information on metabolic surgery provided to students during surgery courses.

**Results:**

The results demonstrate a low level of knowledge on the possibility of applying metabolic surgery to treat morbid obesity. The students themselves expressed a need to improve their knowledge and favorably assessed the proposition of expanding the curriculum to include more information on the subject of metabolic surgery.

**Conclusion:**

The awareness of surgical treatment for morbid obesity among medical students should be improved. The development of an interesting curriculum that is based on current guidelines should be undertaken.

## Introduction

The incidence of obesity has been increasing in recent years not only in Poland but also across all of Europe. It is estimated that obesity affects 10–30 % of women and 10–25 % of men [1]. Obesity is defined with the use of an indicator called body mass index (BMI), which is the ratio of weight in kilograms to the square height in meters. Obesity refers to a BMI above 30 kg/m^2^. In Poland, a BMI > 35 kg/m^2^ is observed in approximately 5 % of the studied population, and a BMI > 40 kg/m^2^ occurs in approximately 1 % of the population [2]. It is a relatively popular conviction that obesity is treated by lifestyle modification, diet therapy, appropriate physical activity, or pharmacological treatment. However, it is worth noting the growing importance of the surgical treatment of obesity, which not only allows for weight reduction but also decreases the risk of death in patients as compared with those treated without surgery [3–5]. The level of knowledge about the possibility of surgical treatment for obesity among general practitioners, who are the medical professionals most aware of the risks of various diseases in patients under their care, remains far from satisfactory [6].

The aim of this study is to determine the level of knowledge of medical students in Poland about the possibility of surgical treatment for obesity, to assess their views on the instruction they have received on this subject during their course of study, and their willingness to improve their knowledge in the future.

Actually, bariatric procedures are performed in 14 centers in Poland. Yearly approximately 2000 bariatric procedures are performed in Poland. All academic centers included in the study have departments offering bariatric procedures.

## Material and methods

The survey was conducted in the years 2012–2013 using an anonymous multiple-choice questionnaire, with 12 questions (one answer permitted for each question) written in collaboration with the President of the Section for Metabolic and Bariatric Surgery of the Society of Polish Surgeons [6]. The first part of the survey was designed to evaluate the knowledge of students. It consisted of questions 1–9, which contained four possible answers, numbered A–D, of which only one is correct. The second part consisted of questions 10–12 and formed the informational part of the survey. Question 1 was devoted to the indications for surgical treatment of obesity, while question 2 consisted of a simple clinical example verifying the students’ ability to adequately qualify patients for bariatric surgery. Question 3 evaluated the students’ awareness of the average incidence of obesity in the Polish general population. Question 4 was designed to check knowledge about the nature of bariatric procedures. In question 5, we asked about their current knowledge as to which method of treatment of obesity is the safest. Questions 6 and 7 are concerned with the impact of bariatric procedures on the overall health of patients who undergo surgery, their life expectancy, and incidence and severity of comorbidities. Question 8 required the students to indicate the procedure that allows for remission in the largest percentage of morbidly obese patients with type 2 diabetes. Question 9 was used to determine the respondents’ knowledge of the term “bariatrics”. Question 10 involved the assessment of the amount of knowledge about bariatric surgery conveyed to medical school students. In Question 11, we asked whether the students knew of bariatric procedures performed in their university clinics, and question 12 asked whether the students would be interested in improving their knowledge on bariatric surgery, for example, through additional optional courses. The full questionnaire is attached as Appendix 1.

The surveys were conducted among sixth-year students of medicine from 4 out of the 11 medical universities in Poland. The chosen schools included the Collegium Medicum, Faculty of Medicine of the Jagiellonian University in Kraków, the First Faculty of Medicine of the Medical University of Warsaw, the Faculty of Medicine of the Karol Marcinkowski University of Medical Sciences in Poznań, and the First Faculty of Medicine of the Medical University of Lublin. At each school, a group of students were randomly selected to complete the survey. Surveys were completed and submitted by 142 students from Kraków, 117 students from Warsaw, 73 students from Poznań, and 136 students from Lublin. In total, there were 468 respondents.

Statistical analysis of the responses was performed with Statistica 6.0 PL software. The following methods of analysis and data presentation were used: a histogram illustrating the number of correct answers at each school, the Fisher–Freeman–Halton test, the Kolmogorov–Smirnov (KS) test to analyze the distribution in the study group, the Mann–Whitney and Kruskal–Wallis test to analyze the significance of differences between average results, and descriptive statistical methods.

## Results

### Knowledge evaluation part

Among the respondents (468 students), the average number of correct answers was 3.34 out of 9, with a standard deviation of 1.77 and a coefficient of variation of 0.4. The most frequently obtained score in the group was 4 correct answers, which was obtained by 117 respondents (25 % of the study group). A total of 47 respondents (10.04 % of the group) failed to mark even one correct answer, while only 4 students (0.85 % of the group) received the maximum number of points.

The obtained score results did not show normal distribution (KS-0.13, *p* < 0.0001). We examined the differences in the average total number of correct answers for each university. The differences were not statistically significant (*p* = 0.1). We also analyzed the value of the coefficient of variation. It was the highest (0.43) among students from the Medical University of Lublin, which means that the school had the largest differences in the level of knowledge between the students. The lowest value of the coefficient of variation (0.27) was observed at the Medical University of Warsaw.

The number of correct responses differed for each question. To assess which questions were the most difficult, we computed an easiness factor, which is the ratio of the number of students who answered a given question correctly to the total number of respondents. By this measure, the easiest were questions 5 and 8, which addressed surgical methods and the relationship between bariatric surgery and diabetes remission. The question that proved to be the most difficult was question 2, which required not only familiarity with indications for surgical treatment of obesity but also the ability to apply them in practice.

### Informational part

#### Analysis of the information about the methods of surgical treatment of obesity provided during the course of studies

When asked about the amount of information about bariatric and metabolic surgery conveyed during the course of studies, 362 students (77.35 % of respondents) evaluated it as insufficient, 40 students (8.54 % of respondents) found the amount of information to be sufficient, and 66 students (14.1 % of respondents) chose the answer “There was no such information taught throughout the course of studies”. None (0 %) of the surveyed students responded that there was too much information about the surgical treatment of obesity taught at their school.

The analysis of the distribution of answers to question 10 showed that the lack of sufficient information was most frequently reported by the students of the Jagiellonian University-Collegium Medicum in Krakow (the answers “insufficient” and “there was no such information taught throughout the course of studies” were marked by a total of 82 % of respondents).

#### Analysis of knowledge about bariatric surgery performed at the university clinic

The number of students who were aware of surgical procedures to treat obesity performed at their university clinic varied significantly, from 80 students (68.37 % of respondents) at the Medical University of Warsaw and 97 students (67.83 %) at the Jagiellonian University Collegium Medicum to 35 students (25.73 %) at the Poznań University of Medical Sciences and 9 students (12.5 %) at the Medical University of Lublin.

#### Additional opportunities to obtain knowledge about the surgical treatment of obesity

The students completing the survey were also asked to comment on the possible introduction of additional opportunities (such as elective courses) to obtain knowledge about metabolic surgery. The idea was viewed favorably by 307 students (65.59 % of the study group), 53 students (11.32 %) were opposed to it, 58 students (12.93 %) considered it unnecessary, and 50 students (10.68 %) indicated that they had no opinion on the subject.

These opinions did not differ significantly between the students of the four participating universities (*p* = 0.13).

## Conclusion

The level of awareness about the surgical treatment of obesity among students from the four surveyed Polish medical universities is not high. We found significant gaps in their knowledge and their ability to apply it in everyday medical practice, which can be attributed to the very limited number of classes devoted to this subject in the curricula of these schools. At the same time, the students indicated a willingness to improve their knowledge of issues related to metabolic surgery, and thus, it is advisable to introduce content on metabolic surgery into the surgery curriculum taught at Polish medical schools.

Results of the study have already inspired changes in the curriculum at the Jagiellonian University in Krakow (from the academic year 2013/2014 2 h lectures at the final year, 2 h seminars at the final year, and 4 h problem-based learning sessions at the final year of education at the Faculty of Medicine are being performed).

## Discussion

It is difficult to compare the results of our research with those obtained by other authors, as (to the best of our knowledge) there are few international publications on the subject, preceding our research.

In 2012, a group of Polish researchers, headed by Martin Giaro published the results of surveys conducted among a group of 778 general practitioners [6]. In 2008, a team from the University of Alexandria in the United States conducted a study of general practitioners with 259 respondents [7]. The group of respondents we analyzed was thus a relatively large cohort (468 respondents). It was also unique in that it was chosen among the population of students in their final year of medical studies.

In the study by Giaro et al. [6], only 8.1 % of the responding general practitioners possessed theoretical knowledge about the indications for surgical treatment of obesity. In the study by Al-Namash Hend, 11.6 % of the surveyed primary care physicians were able to correctly answer questions on the subject [7]. In our survey, 9 % of students had an acceptable level of knowledge about surgical treatment of obesity. It can thus be considered that students of Polish medical schools do not have adequate knowledge about the methods of surgical treatment of obesity.

Although we do not have results of comparable surveys that would analyze the knowledge of medical students from other research centers, we are aware of sporadic reports that refer to the problem of inadequate education of students on the subject of bariatric. An interesting point of reference is provided by the work of Karen D. Marzen-Groller from the United States, who in response to the lack of sufficient knowledge among students at medical schools described in her work, proposed ideas for conducting classes with the application of Case-Based Learning methods [8].

The curricula at Polish medical schools do not reflect sufficient attention to metabolic surgery, or even the widely understood problem of obesity treatment. In 2010, the surgery course schedule at the Medical Faculty of the UJ CM in Kraków did not include a single class on bariatric surgery. The Course Guide of the First Medical Faculty of WUM (Warsaw) does not mention any classes during the fifth and sixth year that are devoted to bariatric surgery. The situation is similar at the Medical University of Lublin. The Medical Faculty of the Poznań University of Sciences is an exception with a class on surgical treatment of obesity included in the surgery course schedule.

The problem with the lack of classes on surgical treatment of obesity has been noted as early as 2001 by teachers of American medical schools [9]. In the same year, the first voices from Europe emerged, advocating the introduction of this subject matter into medical school curricula. However, it was in Israel that classes on bariatric surgery were first introduced into the medical school curricula. In 2005, the Ben-Gurion University of the Negev organized a multidisciplinary curriculum for second-year students devoted to nutrition disorders, which included also classes on bariatric surgery [10, 11]

The results of our study should be cause for concern, as we believe these results imply specific conclusions and recommendations, obesity’s increasing incidence will make the disease a primary challenge for the next generation of medical doctors. We believe that it is crucial to acknowledge that medical students currently lack basic knowledge related to treatment of the disease, and that the medical community should urgently devote attention to this problem.

It is worth noting that medical students in Poland show significant interest in learning about the surgical treatment of obesity, as the present study shows. Close to 66 % of our respondents expressed willingness to participate in additional classes to improve their knowledge on this subject. The group of undecided respondents adds another 10 % of students who could potentially expand their knowledge on bariatric surgery. This opportunity can and should be taken advantage of by the Polish metabolic surgery specialist community with actions to popularize this knowledge among students of medical schools and to develop an interesting curriculum based on current guidelines and modern methods of teaching.

## Appendix

**SURVEY**

1. Which of the following is an indication for the surgical treatment of obesity?
  A. the presence of comorbid conditions of obesity and body mass twice higher than normal
  B. the presence of comorbid conditions of obesity while BMI is above 35
  C. the presence of comorbid conditions of obesity while body is 40 kg higher than normal
  D. none of the above answers is correct
2. Two patients, a man with type 2 diabetes weighing 141 kg at 200 cm of height and a woman with hypertension weighing 80 kg at 150 cm of height reported to a general practitioner. Surgical treatment should be considered in:
  A. the man
  B. the woman
  C. the man and the woman
  D. neither of the above patients
3. Assuming that a typical general practitioner has 2500 patients in their care, how many patients in their practice will meet the indications to be referred to surgical treatment of obesity (choose the most probable answer in the light of epidemiological research carried out in Poland):
  A. 1 patient
  B. 10 patients
  C. 100 patients
  D. 1000 patients
4. Surgical treatment of obesity consists of:
  A. limiting ability to consume or digest and absorb nutrition through surgical operations on the stomach and intestines
  B. removal of excess intra-abdominal fat, from the greater omentum in particular
  C. removal of excess subcutaneous fat within the abdomen and lower limbs, while preserving it in the chest area
  D. introduction of a stomach balloon filled with methylene blue solution or air into the stomach and thus limiting food intake
5. Which of the following techniques of surgery for the treatment of obesity is considered the safest in the light of current medical knowledge?
  A. classic laparotomy—wide opening of the abdominal cavity, enabling an open view of the abdominal cavity
  B. laparoscopic surgery: a minimally invasive technique, also known as keyhole surgery
  C. endoscopic surgery: allowing for access to the abdomen through natural orifices of the body, such as the mouth, anus or vagina
  D. none of the above answers is correct
6. According to the results of research published in the recent years, surgical treatment of obesity in the group of patients undergoing the procedure as compared to patients undergoing conservative treatment results in:
  A. shorter expected survival time
  B. longer expected survival time
  C. longer expected survival time, but lower quality of life
  D. shorter expected survival time, but higher quality of life
7. Surgical treatment of obesity:
  A. causes more frequent occurrence of malignant neoplasms
  B. results in less frequent occurrence of malignant neoplasms
  C. does not impact the frequency of occurrence of malignant neoplasms
  D. has been shown to impact the frequency of occurrence of malignant neoplasms, but there is no sufficient scientific influence to say whether it causes higher or lower frequency of occurrence
8. Which of the surgical methods of treatment of obesity allows for regression in the highest percentage of patients with type 2 diabetes?
  A. laparoscopic gastric sleeve resection
  B. laparoscopic gastric by-pass
  C. laparoscopic gastric banding
  D. endoscopic introduction of a gastric balloon
9. The branch of medicine concerned with treatment of obesity is called:
  A. balneology
  B. lipology
  C. orology
  D. bariatrics
10. Please evaluate the amount of information on metabolic surgery taught throughout the course of your studies.
  A. too much
  B. sufficient
  C. insufficient
  D. there was no such information taught throughout the course of studies
11. Do you know of bariatric surgery procedures performed at the university hospital?
  A. yes
  B. no
12. Please describe your attitude to the possibility of expanding your knowledge on the subject of metabolic surgery through additional courses (for example, optional elective courses) organized by your school.
  A. I would like to participate in such courses.
  B. I am against the organization of such courses.
  C. I think such courses are unnecessary.
  D. I do not have an opinion on this subject.
